# Body condition score prior to parturition is associated with plasma and adipose tissue biomarkers of lipid metabolism and inflammation in Holstein cows

**DOI:** 10.1186/s40104-017-0221-1

**Published:** 2018-01-15

**Authors:** Abdulrahman Alharthi, Zheng Zhou, Vincenzo Lopreiato, Erminio Trevisi, Juan J. Loor

**Affiliations:** 1 Mammalian NutriPhysioGenomics, Department of Animal Sciences and Division of Nutritional Sciences, University of Illinois, 1207 West Gregory Drive, Urbana, IL 61801 USA; 2 Animal and Veterinary Sciences, Clemson University, 146 Poole Agricultural Center, Clemson, SC 29634 USA; 3 Department of Health Science, Interdepartmental Services Centre of Veterinary for Human and Animal Health, Magna Græcia University of Catanzaro, 88100 Catanzaro, Italy; 4 Institute of Zootechnics, Faculty of Agriculture, Food and Environmental Science, Università Cattolica del S. Cuore, 29122 Piacenza, Italy

**Keywords:** Body condition score, Lipid mobilization, Transition cow

## Abstract

**Background:**

Previous research has revealed a strong inflammatory response within adipose (AT) tissue during the transition into lactation. Whether this effect is a result of oxidative stress induced by lipolysis and fatty acid oxidation associated with differences in prepartum body condition score remains to be determined. The objectives of this study were to investigate systemic biomarkers of energy balance and inflammation and the expression of lipid metabolism- and inflammation-related genes in AT during the transition period in dairy cows.

**Results:**

Twenty multiparous Holstein cows were retrospectively divided by body condition score (BCS) prior to parturition into two groups (10 cows/group): BCS ≤ 3.25 (LoBCS) and BCS ≥ 3.75 (HiBCS). Subcutaneous adipose tissue was biopsied from the tail-head region at d − 10, 7 and 20 relative to parturition. Plasma was used to evaluate biomarkers of energy balance (EBAL) [free fatty acids (NEFA), glycerol, insulin] and inflammation [IL-1β, haptoglobin, myeloperoxidase, and reactive oxygen metabolites (ROM)]. Although insulin concentration was not affected by BCS, NEFA was overall greater and glycerol lower in HiBCS cows. Greater activity of myeloperoxidase in plasma coincided with increased haptoglobin and IL-1β postpartum in LoBCS cows. Among genes related with oxidative stress, the expression of the cytosolic antioxidant enzyme *SOD1* was greater in LoBCS compared to HiBCS. Cows in LoBCS compared with HiBCS had greater overall expression of *ABDH5* and *ATGL* along with *ADIPOQ*, indicating enhanced basal lipolysis and secretion of adiponectin. Expression of *CPT1A*, *ACADVL*, and *ACOX1* was greater overall in HiBCS than LoBCS indicating enhanced NEFA oxidation. Although the temporal increase in plasma NEFA regardless of BCS coincided with the profile of *CPT1A*, the gradual decrease in genes related with re-esterification of NEFA (*PCK1*) and glycerol efflux (*AQP7*) coupled with an increase in glycerol kinase (*GK*) suggested some stimulation of NEFA utilization within adipose tissue. This idea is supported in part by the gradual decrease in insulin regardless of BCS. Although expression of the inflammation-related gene toll-like receptor 4 (*TLR4*) was greater in HiBCS versus LoBCS cows at −10 d, expression of *TLR9* was greater in HiBCS versus LoBCS at 20 d. These profiles did not seem to be associated with concentrations of pro-inflammatory biomarkers or ROM.

**Conclusions:**

Overall, data indicated that cows with BCS 3.25 or lower before calving experienced greater alterations in systemic inflammation and basal lipolysis without excessive increases in NEFA plasma concentrations. Despite the greater plasma NEFA around parturition, cows with BCS 3.75 or higher seemed to have a more active system for catabolism of NEFA and utilization of glycerol within adipose tissue. A linkage between those pathways and risk of disorders postpartum remains to be determined.

**Electronic supplementary material:**

The online version of this article (10.1186/s40104-017-0221-1) contains supplementary material, which is available to authorized users.

## Background

During the transition from a non-lactating to a lactating state, dairy cows are susceptible to metabolic disorders and immunologic challenges. Dry matter intake (DMI) decreases in the prepartum and rapidly declines when calving date approaches [1]. Due to the variation between DMI and nutrient requirements, most dairy cows will experience a period of negative energy balance (NEB) [2] that affects metabolism in different tissues, and particularly adipose [3]. Adipose tissue plays an important role in the maintenance of metabolic homeostasis during the transition period [4]. During late pregnancy and early lactation, the adipose tissue starts to break down to generate fatty acids (FA) and glycerol in a process known as lipolysis. The main purpose of lipolysis is to provide energy to other organs in the body. Lipolysis and lipogenesis in adipose tissue are regulated by different hormones around parturition [5]. Non-esterified fatty acid (NEFA) concentration in blood is a good indicator of adipose tissue mobilization [6].

After parturition in dairy cows, the mobilization of fat stored in adipose tissue leads to the loss in body condition score (BCS). Body condition score at calving and early lactation is related to the occurrence of several metabolic disorders like ketosis and fatty liver [7, 8]. It was reported that cows with high BCS at calving lose more body weight and body condition than cows with low BCS [9, 10]. There is a relationship between obesity and oxidative stress in humans [11, 12]. Bernabucci et al. [10] reported a connection between BCS, lipid mobilization and the imbalance in oxidative status in transition cows.

The primary objectives of this study were to evaluate the effect of BCS before calving on plasma and subcutaneous adipose tissue biomarkers of energy balance [free fatty acids (NEFA), glycerol, insulin] and inflammation [IL-1β, haptoglobin, myeloperoxidase, and reactive oxygen metabolites (ROM)] during the transition period in dairy cows retrospectively grouped into a high or low BCS. The subcutaneous depot provides a readily-accessible site for repeated sampling across time, and has been extensively used to understand metabolic mechanisms [4].

## Methods

### Animals and treatments

All the procedures for this study were conducted in accordance with a protocol approved by the Institutional Animal Care and Use Committee of the University of Illinois (Protocol #13023). Twenty Holstein cows (*n* = 10/BCS group) were retrospectively selected according to BCS at calving: high BCS (3.75 ± 0.12; HiBCS) or low BCS (3.25 ± 0.15; LoBCS), based on a 5-point scale [13]. All cows had ad libitum access to the same diet; from −50 to −22 d relative to calving they received a far-off diet (1.40 Mcal/kg of DM, 10.2% RDP, and 4.1% RUP), from −21 d to calving they received a close-up diet (1.52 Mcal/kg of DM, 9.1% RDP, and 5.4% RUP), and from calving until 30 DIM they received a lactation diet (1.71 Mcal/kg of DM, 9.7% RDP, and 7.5% RUP) (Table 1). Diets were fed as a total mixed ration (TMR) once daily (06:30). Dry cows were housed in a free-stall barn with an individual Calan (American Calan, Northwood, NH, USA) gate feeding system. Cows had access to sand-bedded free stalls until 3 d before expected calving date, when they were moved to an individual maternity pen bedded with straw until they calved. After calving, cows were housed in a tie-stall barn and were fed a common lactation TMR in their individual feed bunks once daily in the morning, and milked 3 times daily at approximately 6:00, 14:00, and 22:00.

**Table 1:** Ingredients and chemical composition of experimental diets

	Diet		
Ingredient, % of DM	Far-off	Close-up	Lactation
Alfalfa silage	12.00	8.34	5.07
Alfalfa hay	–	4.29	2.98
Corn silage	33.00	36.40	33.41
Wheat straw	36.00	15.63	2.98
Cottonseeds	–	–	3.58
Wet brewers grains	–	4.29	9.09
Ground shelled corn	4.00	12.86	23.87
Soy hulls	2.00	4.29	4.18
Soybean meal, 48% CP	7.92	2.57	2.39
Expeller soybean meal^a^	–	2.57	5.97
Soychlor^b^	0.15	3.86	–
Blood meal, 85% CP	1.00	–	–
ProVAAl AADvantage^c^	–	0.86	1.50
Urea	0.45	0.30	0.18
Rumen-inert fat^d^	–	–	1.02
Limestone	1.30	1.29	1.31
Salt	0.32	0.30	0.30
Dicalcium phosphate	0.12	0.18	0.30
Magnesium oxide	0.21	0.08	0.12
Magnesium sulfate	0.91	0.99	–
Sodium bicarbonate	–	–	0.79
Potassium carbonate	–	–	0.30
Calcium sulfate	–	–	0.12
Mineral vitamin mix^e^	0.20	0.17	0.18
Vitamin A^f^	0.015	–	–
Vitamin D^g^	0.025	–	–
Vitamin E^h^	0.38	0.39	–
Biotin	–	0.35	0.35

^a^SoyPLUS (West Central Soy, Ralston, IA)

^b^By West Central Soy

^c^Perdue AgSolutions LLC (Ansonia, OH)

^d^Energy Booster 100 (Milk Specialties Global, Eden Prairie, MN)

^e^Contained a minimum of 5% Mg, 10% S, 7.5% K, 2.0% Fe, 3.0% Zn, 3.0% Mn, 5,000 mg of Cu/kg, 250 mg of I/kg, 40 mg of Co/kg, 150 mg of Se/kg, 2,200 kIU of vitamin A/kg, 660 kIU of vitamin D_3_/kg, and 7,700 IU of vitamin E/kg

^f^Contained 30,000 kIU/kg

^g^Contained 5009 kIU/kg

^h^Contained 44,000 kIU/kg

### Adipose tissue

Subcutaneous adipose tissue biopsies were collected from the tail-head region at −10, 7 and 20 d relative to parturition as described by Ji et al. [14]. The samples were immediately frozen in liquid nitrogen and transferred to a −80 °C freezer for future analysis.

### Blood plasma biomarkers

The concentrations of indicators of energy balance including free fatty acids (Cat No. 7 00310, Cayman Chemical Company, Ann Arbor, MI), glycerol (Cat No.10010755, Cayman Chemical Company, Ann Arbor, MI), and insulin (Cat No. 10–1201–01, Mercodia AB, Uppsala, Sweden) were analyzed using commercial kits according to the manufacturer’s protocols. The pro-inflammatory cytokine IL-1β was measured using a bovine ELISA (Cat. No. ESS0027; Thermo Scientific, Rockford, IL). Haptoglobin, myeloperoxidase, and ROM were measured using kits purchased from Instrumentation Laboratory (Lexington, MA) following the procedures described previously using the clinical auto-analyzer (ILAB 600, Instrumentation Laboratory).

### RNA extraction, PCR, and design and evaluation of primers

#### RNA extraction

The frozen tissues were used to extract the RNA using protocols established in our laboratory [15]. Briefly, adipose tissue samples were weighed (~0.2–0.4 g) and immediately placed in 1.2 mL of ice-cold Qiazol reagent (Qiagen 75842; Qiagen Inc., Valencia, CA) for homogenization. After homogenization, genomic DNA was removed from RNA with DNase using RNeasy Mini Kit columns (Qiagen, Hilden, Germany). The Nano-Drop ND-1000 spectrophotometer (Nano-Drop Technologies, Wilmington, DE, USA) was used to measure the concentration of RNA, while the quality of RNA was evaluated using the Agilent Bioanalyzer system (Agilent 2100 Bioanalyzer, Agilent Technologies, Santa Clara, CA, USA). The RNA integrity number averaged 7.33 ± 1.10.

#### qPCR analysis

Primer pairs were designed using the NCBI Primer-BLAST tool, and tested through normal PCR, using the same thermo cycle as the final qPCR analysis, and gel electrophoresis to verify the presence of a single PCR product of the expected size. The product was then purified and sent for sequencing at the University of Illinois Core Sequencing Facility, to assess amplification of the correct target.

The cDNA was synthesized with 100 ng RNA. The RNA was mixed with the Master Mix-1 (MM1) containing 9 μL DNase/RNase free water and l μL random primers (Roche® Cat. No. 11 034 731 001, Roche Diagnostics GmbH, Mannheim, Germany), then incubated at 65 °C for 5 min and kept in ice for 3 min. The reaction was performed using the Eppendorf Mastercycler®. Nine  μL of Master Mix-2 (MM2) consisting of 1.625 μL DNase/RNase free water, 4 μL 5× First-Strand Buffer, 1 μL Oligo dT18, 2 μL 10 mmol/L dNTP mix (10 mmol/L; Cat. No. 18427–088; Invitrogen), 0.25 μL of Revert aid (200 IU/μL; Cat. No. EP 0441; Fermentas), and 0.125 μL of RNase inhibitor (20 U/μL; Cat. No. EO 0382; Fermentas). Samples were then incubated (MM1 + RNA and MM2) at the following temperature program: 25 °C for 5 min, 42 °C for 60 min and 70 °C for 5 min. An aliquot of undiluted cDNA from all samples was pooled to make the standard curve by diluting with DNase/RNase free water, then the cDNA was diluted 1:4 with DNase/RNase free water.

Quantitative PCR was performed using 4 μL diluted cDNA combined with 6 μL of a mixture containing 5 μL 1 × SYBR Green master mix (Applied Biosystems, CA, USA), 0.4 μL each of 10 μmol/L forward and reverse primers, and 0.2 μL DNase/RNase free water in a MicroAmp™ Optical 384-Well Reaction Plate (Applied Biosystems, CA, USA). An ABI prism 7900 HT SDS instrument was used at the following temperature program: 2 min at 50 °C, 10 min at 95 °C, 40 cycles of 15 s at 95 °C, and 1 min at 60 °C. The presence of a single PCR product was verified by the dissociation protocol using incremental temperatures to 95 °C for 15 s plus 65 °C for 15 s. Gene symbols, ID, and accession numbers of PCR primers are reported in Additional file 1: Table S1. The sequencing products were validated through BLASTN at the National Center for Biotechnology Information (NCBI) database (Additional file 1: Table S2).

The final data were normalized using the geometric mean of three internal control genes: *UXT*, *GAPDH* and *RPS9* previously validated by our group [16, 17]. The relative mRNA abundance was calculated as previously reported [18] using the median ∆Ct (∆Ct = Ct of the gene – geometrical mean Ct of internal control genes) corrected by efficiency (E), where % relative mRNA abundance = [1/E^∆Ct^] / ∑[1/E^∆C^] all measured genes × 100 (Additional file 1: Table S3). The PCR efficiency was calculated for each gene using the standard curve method [E = 10^(−1/slope)^ ].

#### Statistical analysis

After data normalization with the geometric mean of the internal control genes, the quantitative PCR data were log_2_ transformed before statistical analysis to obtain a normal distribution. Log_2_ transformed data were subjected to ANOVA and analyzed using repeated measures in PROC MIXED of SAS (SAS Institute, Inc., Cary, NC, USA). The statistical model included Day (−10, 7 and 20 d relative to parturition), BCS (HiBCS and LoBCS), and their interactions (BCS × Day) as fixed effects, and cow within BCS group as random effect. The analysis of DMI (carried out separately for pre- and post-calving periods), milk yield, and plasma biomarker data was performed with the same model for gene expression analysis. For DMI and milk yield the time effect included all d within each period (from −22 to −1 d for pre-calving, and 1 to 30 d for post-calving). Furthermore, BCS data (as units of change between – 3 and 3 wk relative to parturition) were analyzed with the PROC MIXED of SAS with BCS group as fixed effect. Data were considered significant at *P* ≤ 0.05 and tendencies at *P* ≤ 0.15.

## Results

### Dry matter intake, milk production, and BCS

**Fig. 1:**
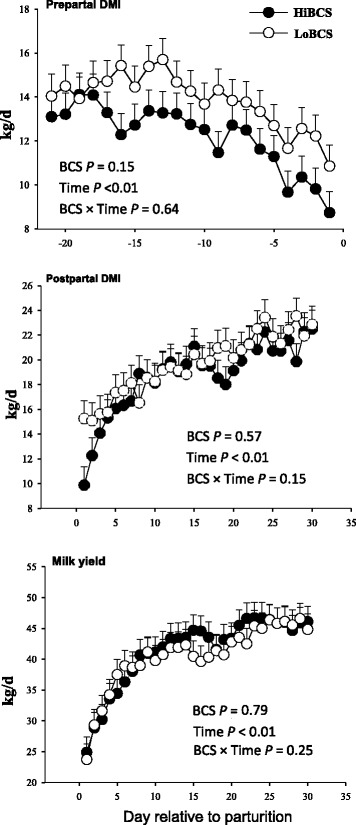
Daily dry matter intake from −21 through 30 d around parturition (top panels), and daily milk yield (kg/d) (least squares mean ± SEM) in cows with high body condition score (HiBCS) or low body condition score (LoBCS) at parturition

**Fig. 2:**
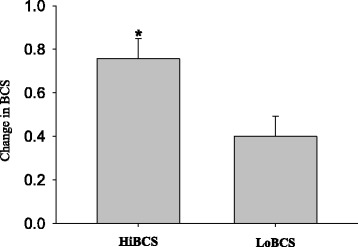
Change in body condition score (BCS) units between −3 and 3 wk relative to parturition in cows with high body condition score (HiBCS) or low body condition score (LoBCS) at parturition. *Groups differ (*P* ≤ 0.05)

There was no overall effect of BCS on milk yield or DMI (Fig. 1). However, the HiBCS group experienced a higher loss of BCS than the LoBCS group (*P* < 0.01; Fig. 2) between −3 and 3 wk relative to calving.

### Plasma biomarkers of energy balance

**Fig. 3:**
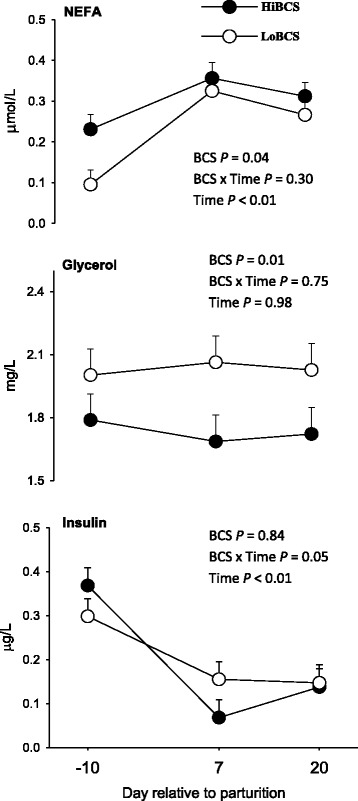
Plasma concentrations of indicators of energy balance (free fatty acids, NEFA; glycerol; insulin) in cows with high body condition score (HiBCS) or low body condition score (LoBCS) at parturition. *Means between groups differ (BCS × Day *P* ≤ 0.05)

The concentration of free fatty acids (NEFA) was greater overall for HiBCS cows, particularly due to differences at −10 d from parturition (Fig. 3). In contrast, concentrations of glycerol were fairly stable around parturition but were lower in HiBCS cows. There was a BCS × Day effect (*P* = 0.05) for concentration of insulin namely due to the decrease in concentration at 7 and 20 d postpartum compared with −10 d.

### Plasma biomarkers of inflammation and oxidative stress

**Fig. 4:**
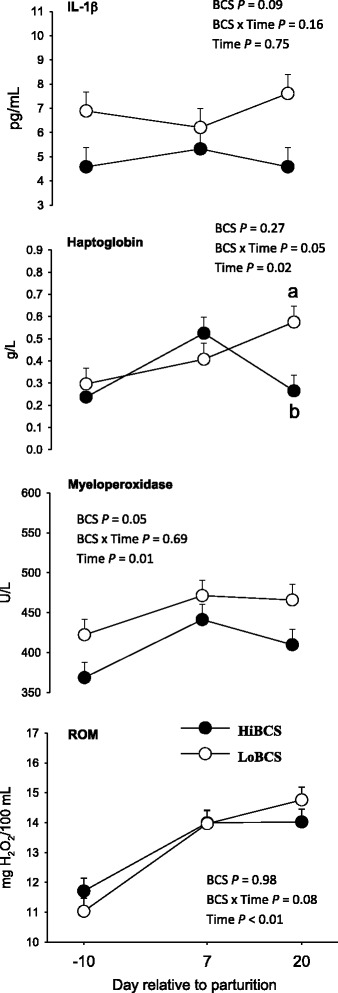
Plasma concentrations of indicators of inflammation (IL-1β, haptoglobin, and myeloperoxidase) and oxidative stress (reactive oxygen metabolites, ROM) energy balance (free fatty acids, NEFA; glycerol; insulin) in cows with high body condition score (HiBCS) or low body condition score (LoBCS) at parturition. ^a,b^Means between groups differ (BCS × Time *P* ≤ 0.05)

The concentration of the positive acute-phase protein haptoglobin had a BCS × Day effect (*P* = 0.05) namely due to a gradual increase in concentration at 20 d for LoBCS cows compared with a gradual decrease for HiBCS cows (Fig. 4). This response in LoBCS cows mirrored the activity of myeloperoxidase, an enzyme secreted by activated immune cells (e.g. neutrophils), which was greater overall in LoBCS cows. The concentration of the pro-inflammatory cytokine IL-1β was fairly constant around parturition, and tended to be greater in LoBCS cows (*P* = 0.09). No effect of BCS (*P* = 0.98) was detected for concentration of ROM, but there was a gradual increase between −10 and 20 d around parturition.

### Genes involved in lipolysis and adipokine synthesis

**Fig. 5:**
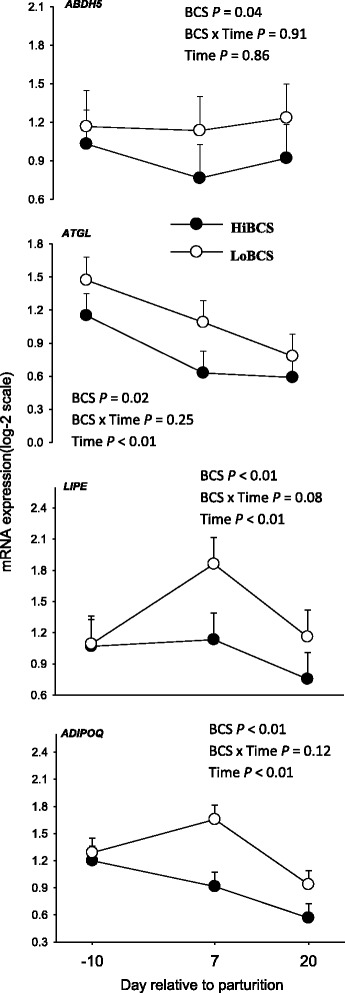
mRNA expression (least squares mean ± SEM) of genes involved in basal (*ABDH5, ATGL*) and stimulated (*LIPE*) lipolysis and the anti-lipolytic adipokine adiponectin (*ADIPOQ*) lipolysis in cows with high body condition score (HiBCS) or low body condition score (LoBCS) at parturition

The expression of *ATGL* (which catalyzes the first step in triglyceride hydrolysis) and *LIPE* was affected by BCS (*P* < 0.05), with *ATGL* having greater overall expression in LoBCS than HiBCS cows (Fig. 5). There was a strong tendency for an interaction effect on expression of *LIPE* (BCS × Day, *P* = 0.07) due to upregulation at d 7 and 20 in LoBCS than HiBCS cows. An overall greater expression of *ABDH5* (*P* = 0.04) was detected in LoBCS cows. The gene encoding the adipokine adiponectin (*ADIPOQ*) was affected by BCS due to greater expression in LoBCS than HiBCS cows (*P* ≤ 0.05).

### Fatty acid oxidation and transport genes

**Fig. 6:**
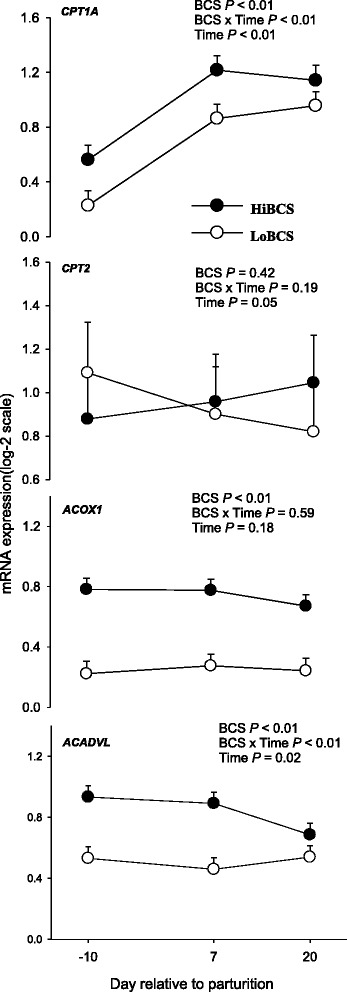
mRNA expression (least squares mean ± SEM) of genes involved in fatty acid oxidation (*CPT1A, CPT2, ACOX1, ACADVL*) in cows with high body condition score (HiBCS) or low body condition score (LoBCS) at parturition. *Means between groups differ (BCS × Day *P* ≤ 0.05)

There was an interaction (BCS × Day, *P* ≤ 0.05) for *CPT1A* (a key enzyme in fatty acid oxidation) due to greater expression at −10 and 7 d in HiBCS compared with LoBCS cows (Fig. 6). There was no BCS or BCS × Day effect for the expression of *CPT2*. However, HiBCS cows experienced a gradual increase (time, *P* = 0.05) between −10 and 20 d. Acyl-CoA dehydrogenase very long chain (*ACADVL*) (also involved in oxidation of long chain fatty acids) was greater in HiBCS than LoBCS cows across time (*P* ≤ 0.05). There was a significant interaction (BCS × Day *P* ≤ 0.05) due to higher expression at −10 and 7 d in HiBCS than LoBCS cows. Similar to *ACADVL*, there was a BCS (*P* ≤ 0.05) effect for *ACOX1* due to greater overall expression in HiBCS cows.

### Transporter and fatty acid binding protein gene expression

**Fig. 7:**
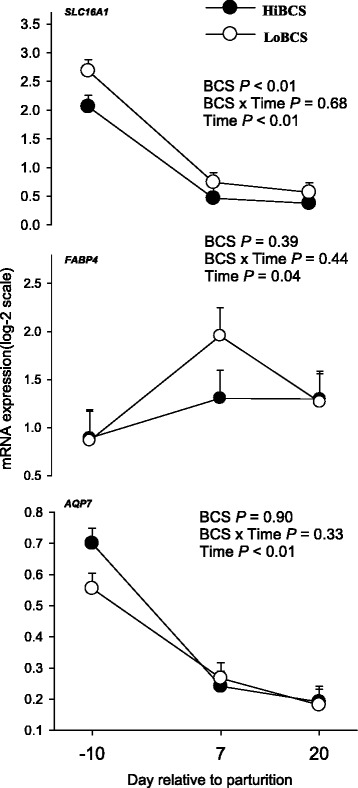
mRNA expression (least squares mean ± SEM) of genes involved in monocarboxylic acid transport (*SLC16A1*), intracellular fatty acid transport (*FABP4*), and glycerol transport (*AQP7*) in cows with high body condition score (HiBCS) or low body condition score (LoBCS) at parturition

The expression of solute carrier family 16 member 1 (*SLC16A1;* Fig. 7), involved in short-chain fatty acid transport, was affected by BCS (*P* ≤ 0.05) due to greater expression in LoBCS than HiBCS cows. We observed a Day effect (*P* = 0.04) for *FABP4* due to higher expression in both LoBCS and HiBCS groups at 7 d (Fig. 5). The expression of the glycerol transporter *AQP7* was not affected by BCS or BCS × Day, but there was a Day effect (*P* ≤ 0.05) due to a decrease in expression in both groups between −10 and 20 d (Fig. 7).

### Glyceroneogenic gene expression

**Fig. 8:**
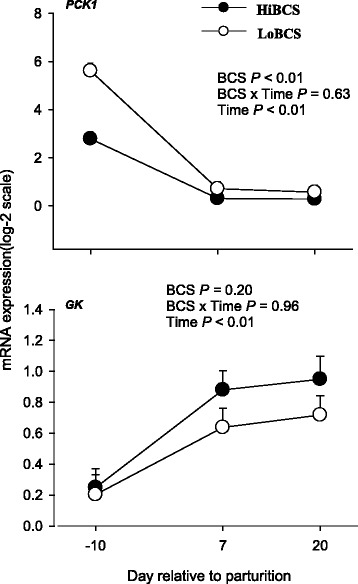
mRNA expression (least squares means ± SEM) of genes involved in glycerol-3-phosphate synthesis (phosphoenolpyruvate carboxykinase 1, *PCK1*; glycerol kinase, *GK*) in cows with high body condition score (HiBCS) or low body condition score (LoBCS) at parturition

The expression of *PCK1* (which plays an important role in glyceroneogenesis) was overall greater in LoBCS (*P* < 0.01) than HiBCS cows (Fig. 8). However, both groups had higher *PCK1* expression (*P* < 0.01) in the pre-calving compared with post-calving period. Glycerol kinase (*GK*) was not affected by BCS, but there was a Day effect (*P* ≤ 0.05) due to an increase in expression at 7 and 20 d compared with −10 d in both HiBCS and LoBCS.

### Inflammation and oxidative stress gene expression

**Fig. 9:**
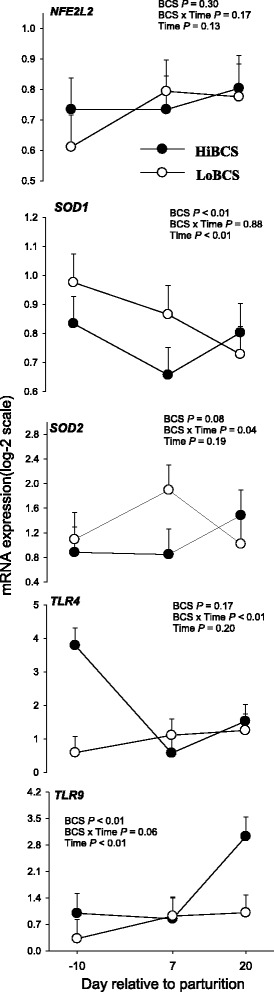
mRNA expression (least squares means ± SEM) of genes involved in inflammation and oxidative stress in cows with high body condition score (HiBCS) or low body condition score (LoBCS) at parturition. *Means between groups differ (BCS × Day *P* ≤ 0.05)

The expression of *NFE2L2* (a transcription regulator involved in oxidative stress and inflammation) was not affected by BCS, Day or their interaction (Fig. 9). We detected a BCS effect (*P* ≤ 0.05) for *SOD1* due to higher expression in LoBCS cows at −10 and 7 d. There was an interaction (BCS × Day, *P* ≤ 0.05) observed for the mitochondrial enzyme *SOD2* because of greater expression at d 7 in LoBCS than HiBCS cows. Although Toll-Like Receptor 4 (*TLR4*) was not affected by BCS or time, we observed an interaction resulting in higher expression at −10 d in HiBCS cows. We detected a BCS effect for the expression of *TLR9* (*P* ≤ 0.05) because of an increase in the expression at 20 d in HiBCS compared with LoBCS cows.

## Discussion

Around calving, adipose tissue becomes active by increasing the mobilization of body fat reserves to provide energy to other tissues. Adipose triglyceride lipase (ATGL) is the rate-limiting enzyme of lipolysis and is upregulated during fasting partly through transcription [19]. The degradation of triacylglycerol (TAG) is regulated by *ATGL* and *LIPE,* the two key enzymes in basal and stimulated lipolysis [20]. The complete activation of *ATGL* requires binding of the protein ABHD5 which is the activator of *ATGL* [14]. Hormone sensitive lipase (LIPE) is able to hydrolyze TAG, with the activation of LIPE occurring via cyclic AMP as a result of beta-adrenergic stimulation (i.e. post-translational regulation) [21]. Thus, although opposite to what would be expected, the greater expression of *ATGL*, *LIPE* and *ABDH5* in LoBCS compared with HiBCS cows indicated a greater state of basal and stimulated lipolysis over time. Clearly, cows with HiBCS would be expected to have accumulated more fat depots and potentially have greater lipolytic activity partly through the action of *ATGL* and *LIPE* [14]. Although those cows had greater plasma NEFA primarily prior to calving, despite the gradual increase in NEFA postpartum the glycerol concentration was lower throughout the transition period. Thus, alternate mechanisms within adipose tissue seem to account for differences between lipolytic marker genes and systemic indicators.

In the context of energy balance around parturition, the greater expression of *ADIPOQ* in the LoBCS cows is not intuitive because in non-ruminant obese subjects concentration of adiponectin in the circulation is lower due in part to a decrease in transcription [22]. It could be speculated that a lower circulating concentration of adiponectin in obese states or a high BCS triggers a response within adipose tissue to upregulate *ADIPOQ*, hence, its availability to peripheral tissues. Overall, the *ADIPOQ* data agree with a previous report of a negative correlation between BCS and serum adiponectin concentration [23].

Both the adipose tissue sensitivity to insulin and its concentration affect the degree of lipolysis and lipogenesis [24]. Data from rats and humans demonstrated that late-pregnancy is an insulin-resistant state [25]. The greater mRNA expression of *CPT1A*, *ACADVL* and *ACOX1* (genes involved in FA oxidation) in HiBCS cows indicated an increase in the use of NEFA as energy sources within adipose tissue, either through mitochondrial or peroxisomal β-oxidation. At least in non-ruminants, the activity of these enzymes is partly regulated via changes in transcription [26]. Despite the increase in FA release from adipose tissue after calving regardless of BCS, ensuring that tissues like liver or muscle could oxidize them for energy [27], the present data underscore a capacity of adipose for utilization of endogenously-released FA. Furthermore, the greater lipolytic gene expression indicated that LoBCS cows likely were mobilizing more TAG than HiBCS but at the same time they did not seem to have the ability to increase the utilization of these FA through oxidation. Because in non-ruminant cells solute carrier family 16 member 1 (*SLC16A1*) is controlled through transcription [28], we speculate that the upregulation of in LoBCS cows might have increased uptake of short chain monocarboxylates such as pyruvate, lactate and/or volatile fatty acids. As such, adipocytes would not have required increased oxidation of FA to generate energy.‬‬‬‬‬

Aquaglyceroporin aquaporin-7 (*AQP7*) is highly-expressed in non-ruminant adipose tissue, and helps facilitate the efflux of glycerol that is released from adipose tissue during lipolysis. A reduction in transcription of *AQP7* is related to TAG accumulation in adipose tissue [29] and a lower plasma concentration of glycerol under fasting and fed conditions [30]. The sharp decrease in *AQP7* expression between prepartum and postpartum agrees with the only published study in periparturient dairy cows [31], but was unrelated with plasma glycerol concentrations which were stable around parturition. The fact that bovine adipocyte size and *AQP7* mRNA expression are negatively correlated [31] agrees with observations from McNamara [4] demonstrating a marked reduction in adipocyte size after calving, and also data from AQP7-null mice in which body fat mass increased as a result of adipocyte hypertrophy [32]. Despite the lack of association between AQP7 and plasma glycerol, the available data in dairy cows seem to indicate a functional role for AQP7 in adipocyte biology. It could be possible that plasma glycerol concentration is partly regulated by its use in the liver during gluconeogenesis [6].

Transcription of *PCK1* is related to glyceroneogenesis (i.e. de novo synthesis of glycerol-3-phosphate for TAG production) in non-ruminants [33], and we speculate that the greater expression of *PCK1* in LoBCS cows could have been a response to help re-esterify FA released as a result of greater *ATGL* and *LIPE* in those cows. Mechanistically, such response would make sense given that a high rate of FA recycling during lipolysis can help maintain normal intracellular concentrations of FA when enough energy has been generated through oxidation [33]. It is possible that recycling of the excess amount of FA that were hydrolyzed after calving in LoBCS cows was partly regulated by hormonal signals (e.g. epinephrine) and the upregulation of glycerol kinase (GK) (a transcriptional target of PPARGC-1α in non-ruminant adipocytes) as a way to maintain TAG stores in those cows. Comparing −3 wk versus 3 wk relative to parturition reveals that HiBCS cows lost more body condition, which supports the role of *PCK1* in maintaining TAG stores.

Oxidative stress, resulting in the increase in ROM production, can cause alterations of cell membranes and changes in cellular function [34, 35]. The enzyme superoxide dismutase (SOD) catalyzes an antioxidant mechanism that decreases concentration ROM, hence, plays an important role in maintenance of proper antioxidant capacity in tissues [36]. It was previously reported that cows with high BCS before calving and with more BCS losses had lower SOD activity and higher ROM in the circulation [10]. Furthermore, several studies in humans linked obesity with higher oxidant and lower antioxidant concentrations [37, 38]. Because a previous study detected that the inhibition of *SOD2* expression caused accumulation of ROM [39], we speculate that the greater expression of *SOD1* in LoBCS cows could have helped the adipose tissue maintain a proper antioxidant status. Although plasma ROM did not differ due to BCS, concentrations increased gradually during the transition period and is unknown if they could affect adipose tissue. From a mechanistic standpoint, however, it could be possible that the greater *ATGL* and *LIPE* in LoBCS cows could have generated more FA and greater concentrations of ROM.

Toll-like receptors are essential in the defense mechanism against microbes and activate the innate immune response during inflammation [40]. Greater overall expression of *TLR9* in HiBCS compared with LoBCS and greater expression of *TLR4* at day −10 in the same group of cows indicated a more pronounced localized state of inflammation. Toll-like receptor 4 binds to bacterial lipopolysaccharide which is the main component of all Gram-negative bacteria [41]. Also, *TLR4* could be activated via saturated fatty acids [42] and such response could be a reason for the increase in expression of *TLR4* in HiBCS. Because the systemic concentrations of inflammatory biomarkers indicated a more pronounced response in LoBCS cows, it would seem that the adipose tissue depot has an independent mechanism (at least from the transcriptional standpoint) to control this process. The identity of such mechanisms are unclear; however, a recent study in mice demonstrated that obesity is associated with the release of cell-free DNA (cfDNA) which could stimulate the resident macrophages via the *TLR9* pathway [43]. Further research could help demonstrate if the same linkage exists in dairy cow adipose tissue.

## Conclusions

The greater expression of genes associated with lipolysis in LoBCS cows indicated a greater state of basal lipolysis in this group, and at the same time those cows had higher expression of *PCK1*, which indicated more re-esterification of FA to maintain TAG stores. The greater expression of genes involved in FA oxidation in the HiBCS indicated a higher (and potentially more efficient) use of FA as energy substrates within adipose tissue. However, the greater expression of *TLR4* and *TLR 9* in HiBCS cows and the lower expression of *SOD* in the same group could be associated with the observed higher loss of BCS postpartum. Whether this inflammatory status within adipose tissue elicits positive or negative effects remains to be established.

## Additional file

Additional file 1: Table S1. Gene accession number, symbol, and forward and reverse sequences. **Table S2.** Sequencing results obtained from PCR products. **Table S3.** qPCR performance of genes measured in subcutaneous adipose tissue. (DOCX 19 kb)

## Abbreviations

BCS: body condition score
DMI: dry matter intake
FA: fatty acid
HiBCS: high BCS
LoBCS: low BCS
NEFA: free fatty acid
QPCR: quantitative polymerase chain reaction
RIN: RNA integrity number
TMR: total mixed ration
