# Supplementary material for: Body condition score prior to parturition is associated with plasma and adipose tissue biomarkers of lipid metabolism and inflammation in Holstein cows
Source: J Anim Sci Biotechnol. 2018 Jan 15;9:12. doi: 10.1186/s40104-017-0221-1 (PMC5775576; doi:10.1186/s40104-017-0221-1)
Supplement: Additional file 1: Table S1. — Gene accession number, symbol, and forward and reverse sequences. Table S2. Sequencing results obtained from PCR products. Table S3. qPCR performance of genes measured in subcutaneous adipose tissue. (DOCX 19 kb) [file 40104_2017_221_MOESM1_ESM.docx]

**Table S1.** Gene accession number, symbol, and forward and reverse sequences.

| **Accession Number** | **Gene** | **Forward Sequence** | **Reverse Sequence** |
| --- | --- | --- | --- |
| **NM_001076063.1** | *ABDH5* | CTGCAGATGATGTGGGAAAGC | GACTGCCTGGTTCTCGTGTCA |
| **EF140760.1** | *LIPE* | TCAGTGTCCAAGACAGAGCCAAT | CATGCAGCTTCAGGCTTTTG |
| **XM_005204534** | *CPT2* | ATCATTTCTTTCACTCTGCAGAACA | GGAGGCCTGAGGGAGTCATT |
| **XM_005201085.1** | *SOD1* | GGCTGTACCAGTGCAGGTCC | GCTGTCACATTGCCCAGGT |
| **NM_001046005.1** | *ATGL* | CACCAGCATCCAGTTCAACCT | CTGTAGCCCTGTTTGCACATCT |
| **NM_174314** | *FABP4* | TGGTGCTGGAATGTGTCATGA | TGGAGTTCGATGCAAACGTC |
| **NM_201527.2** | *SOD2* | TGTGGGAGCATGCTTATTACCTT | TGCAGTTACATTCTCCCAGTTGA |
| **XM_005227376** | *CPT1A* | TCGCGATGGACTTGCTGTATA | CGGTCCAGTTTGCGTCTGTA |
| **NM_001076378** | *ACADVL* | TGCTGCTAACCGTACCCAGTTT | CGCCATGGACTCAGTCAGTCACAT |
| **NM_001035289.2** | *ACOX1* | ACCCAGACTTCCAGCATGAGA | TTCCTCATCTTCTGCACCATGA |
| **NM_001076378.2** | *AQP7* | ATTGTGACTGGCATCCTTG | TGGTTCTGAAGACTTGTGAG |
| **BC140488** | *ADIPOQ* | GATCCAGGTCTTGTTGGTCCTAA | GAGCGGTATACATAGGCACTTTCTC |
| **NM_174198.6** | *TLR4* | TAGTTAAAGCTCAGGTCCAGCATCT | TAGTTAAAGCTCAGGTCCAGCATCT |
| **NM_183081** | *TLR9* | GCCAAGCATCCTTCCCTG | GCACCAGGAGAGAAAGGG |
| **NM_174737** | *PCK1* | AAGATTGGCATCGAGCTGACA | GTGGAGGCACTTGACGAACTC |
| **NM_001037319.1** | *SLC16A1* | CCTGTGGGACTGAAGGGTAAAT | ATGATTCCCACAGAAATGTCCAGTAT |
| **NM_011011678.2** | *NFE2L2* | GGGAATATCAGGAACAAGTGATTGA | AGCAGATGATTTGTACTTCGATGACT |
| **NM_001075236** | *GK* | AAAGCTCCGAGGAAATTGAAAAAC | GTGCATACAGCCCCGAAAAT |

**Table S2.** Sequencing results obtained from PCR products.

| **Primer Name** | **Sequence** |
| --- | --- |
| *ABHD5* | CGTCCACCTCTCGCGTATTACCGCTCTGGGACGCAGAGTAAGGGAATCTGACACGAGAACCAGGCAGTTAA |
| *LIPE* | AGCAGCCCTGACCCGGCCGGAGGGCTCACTGGGAACCGACTCCCTCAAAAGCCTGAAGCTGCATGAA |
| *CPT2* | GCATAGCGTTGATGAGACACCTCCGGGGTGCCTTAGGCCATATTTTATTGATGACCCTGAAGAAGCCTGGAGTAC AAT |
| *SOD1* | GCGTGCACGGGCAGGGGTGCTGTTCAGTGACGGTGGCACGACCTTCCTACAGAACTTCAAAGGTGATTTCGTGA |
| *ATGL* | GCCTCGCCTTCAGGCCTGTTCCGCCCGAGCCCTGGTNCTTCGAGAGATGTGCAAACAGGGCTACAGAACCC |
| *FABP4* | CCCGAGTTATGAGAGAGCGTAGCCAAGGGATATTGAAATGGATGACGTTTGCATCGAACCTCCAAA |
| *SOD2* | GCATGTTTGGCCGATTATCTGAGGCCATTTTGGAATGTGATCAACTGGGAGAATGTAACTGCAATAC |
| *CPT1A* | GGACTATGAAGGTAAACCAGGCCCGGGACGCCCTTCGTACAGGCCTCTCGCTCCAGCTGGCTCATTACAAGGGAC CA |
| *AQP7* | ATTGTGACTGGCATCCTTGTTGTCATCATCGGAATATCCCTGGGCATGAACTCAGGATATGCCATCAACCCATCCCGGGACCT |
| *ACADVL* | GAAACTAACTTTGTGGCGTATCCAGGAGAAGCGTGCCCGGAAGTGCTATGCTGCAGTATGTGACTGAGAATCCAT GGCGA |
| *ACOX1* | ATCCTCGTATCCGCGTTCAGGGTGCGTTTAAGAAGAGTGCCATCATGGTGCAGAAGATGAGGAAATCCCC |
| *ADIPOQ* | GGCAGTGGTAACTGGATCACTGGGATCGAGGTCCCCGAGGCTTTCCAGGAACCCCAGGCAGAAAGGGAGAACCT GGAGAAAGTGCCTATGTATACACGCTCTATG |
| *TLR4* | GCATCCCTCACCGTTATGGTCAGGTGAATTCCTGGGATAAGGCCAGGCTTCCTCTTGTTGGTTACTTCAGCCAGAAA |
| *TLR9* | GGCACGGGAAGTGGGCGCCAAGCATCCTTCCCTGCAGCTGCCTCCCAACCTGCCCGCCAGACCCTCTGGAGAAGCCGCATTCCCTGTCATGGGCCCCTACTGTGCCCCGCACCCCCTTTCTCTCCTGGTGC |
| *PCK1* | GCCATGTGTACAGCAGTCGCATCATGACGAGGATGGGCACCAGCGTCCTGGAAGCGCTGGGGGACGGCGAGTTCG TCAAGTGCCTCCACAAA |
| *SLC16A1* | GCACTCGTCACCAATATTCTATGTGGCCTGGGTGATCCTACCAGGTGGGTGCCTCAGGTGCAAATACCTGGACATT TCTGTGGGAATCATATGAA |
| *GK* | TACTTCTTATGGCTGCTATTTCGTCCCAGCATTTTCGGGGGCTGTATGCACAAA |

**Table S3.** qPCR performance of genes measured in subcutaneous adipose tissue.

| **Gene** | **Median Ct^1^** | **Median ∆Ct^2^** | **Slope^3^** | **(R^2^)^4^** | **Efficiency^5^** |
| --- | --- | --- | --- | --- | --- |
| *ABDH5*  *HSL*  *HSL* | 25.48 | 3.84 | -3.19 | 0.99 | 2.06 |
| *LIPE* | 17.87 | -3.61 | -3.14 | 0.99 | 2.08 |
| *CPT2* | 23.89 | 2.24 | -3.20 | 0.99 | 2.05 |
| *SOD1* | 21.50 | -0.06 | -3.66 | 0.97 | 1.88 |
| *ATGL* | 19.30 | -2.38 | -3.42 | 0.97 | 1.96 |
| *FABP4* | 15.12 | -6.45 | -3.13 | 0.98 | 2.09 |
| *SOD2*  *CPT1A* | 23.17 | 1.84 | -3.56 | 0.97 | 1.91 |
| *CPT1A* | 23.61 | 1.77 | -3.11 | 0.99 | 2.10 |
| *AQP7* | 23.20 | 1.56 | -3.67 | 0.99 | 1.87 |
| *ACADVL* | 22.74 | 1.13 | -3.09 | 0.99 | 2.09 |
| *ACOX1* | 22.22 | 0.66 | -3.06 | 0.97 | 2.12 |
| *ADIPOQ* | 18.70 | -2.65 | -3.33 | 0.99 | 2.00 |
| *TLR4*  *TLR-9* | 29.83 | 8.67 | -3.05 | 0.99 | 2.12 |
| *TLR9* | 28.46 | 7.07 | -3.02 | 0.99 | 2.15 |
| *PCK1* | 27.05 | 5.30 | -3.23 | 0.99 | 2.04 |
| *SLC16A1* | 23.73 | 1.92 | -3.19 | 0.99 | 2.06 |
| *NFE2L2* | 19.96 | -1.47 | -3.04 | 0.98 | 2.13 |
| *GK* | 26.56 | 5.10 | -3.46 | 0.99 | 1.94 |

^1^The median is calculated considering all time points and all cows.

^2^The median of ∆Ct is calculated as [Ct gene – geometrical mean of Ct of 3 internal control genes] for each time point and each cow.

^3^Slope of the standard curve.

^4^R^2^ stands for the coefficient of determination of the standard curve.

^5^Efficiency is calculated as [10^(-1/slope)^].
